# β-defensin 2 as an Adjuvant Promotes Anti-Melanoma Immune Responses and Inhibits the Growth of Implanted Murine Melanoma *In Vivo*

**DOI:** 10.1371/journal.pone.0031328

**Published:** 2012-02-13

**Authors:** Han-fang Mei, Xiao-bao Jin, Jia-yong Zhu, Ai-hua Zeng, Qiang Wu, Xue-mei Lu, Xiao-bo Li, Juan Shen

**Affiliations:** 1 Guangdong Provincial Key Laboratory of Pharmaceutical Bioactive Substances, Guangdong Pharmaceutical University, Guangzhou Higher Education Mega Center, Guangzhou, China; 2 School of Basic Sciences, Guangdong Pharmaceutical University, Guangzhou Higher Education Mega Center, Guangzhou, China; 3 Department of Biochemistry and Molecular Biology, Guangdong Pharmaceutical University, Guangzhou Higher Education Mega Center, Guangzhou, China; 4 Institute of Tropical Medicine and Public Health, Southern Medical University, Guangzhou, China; Institut Pasteur, France

## Abstract

β-defensin 2 is a small antimicrobial peptide of the innate immune system and has been thought to regulate anti-tumor immunity. However, little is known on whether β-defensin 2 could modulate melanoma-specific NK and T cell responses. In this study, we first cloned the murine β-defensin 2 gene by RT-PCR and generated the β-defensin 2 stably expressing B16 cells (B16-mBD2). Subsequently, we evaluated whether vaccination with irradiated B16-mBD2 could modulate the growth of implanted B16 cells and determined the potential mechanisms underlying the action of B16-mBD2 vaccine in modulating the growth of B16 tumors in C57BL/6. We found that vaccination with irradiated B16-mBD2, but not with control B16-p or parental B16, inhibited the development and progression of B16 tumors, and prolonged the survival of tumor-bearing mice. However, vaccination with irradiated B16-mBD2 failed to inhibit the development of B16 tumors in the CD4^+^- or CD8^+^-depleted recipients. Furthermore, vaccination with irradiated B16-mBD2 stimulated strong NK activity and promoted potent B16-specific CTL responses, accompanied by augmenting IFN-γ and IL-12, but not IL-4, responses in the recipient mice. Moreover, vaccination with irradiated B16-mBD2 promoted the infiltration of CD8^+^ and CD4^+^ T, NK cells and macrophages in the tumor tissues. These data suggest β-defensin 2 may act as a positive regulator, promoting anti-tumor NK and T cell responses *in vivo*. Therefore, β-defensin 2 may be used for the development of immunotherapy for the intervention of melanoma.

## Introduction

Malignant melanoma is derived from melanocytes and its incidence is currently increasing worldwide [1]. Although melanoma is thought to be resistant to a wide range of systemic chemotherapeutic agents, it is considered to be an immunogenic tumor [2]. Hence, melanoma has become an excellent target for anti-tumor immunotherapy. Many studies have attempted for the development of vaccination, and previous studies have shown promising efficacy of melanoma-based vaccines in inhibiting the growth of melanoma cells in a number of murine melanoma models [3], [4]. However, the results of clinical trials are disappointed, and currently, there is no available vaccine with a clinically proven efficacy against melanoma [2], [5], [6]. Design of new effective vaccines for melanoma at clinic is currently one of the research focuses in the anti-melanoma field. Theoretically, a vaccine for inducing immune responses should depend on its immunogenicity. Given the low immunogenicity of tumors, the efficacy of tumor-based vaccines may depend not only on the type of vaccines, but also on an ideal adjuvant for augmenting strong anti-tumor T cell immunity [2]. However, currently available adjuvants used in humans are unable to induce potent T cell immunity. Therefore, the discovery of new adjuvants for the development of vaccines for the immunotherapy of tumors will be of great significance.

Antimicrobial peptides are ancient arms of the innate immune response in all life forms, and many of them can directly neutralize invading microbes. Defensin is a family of small (3.5–4.5KD) cationic antimicrobial peptides and widely distributed [7]. Recent studies have demonstrated that defensins can modulate immune responses [7], [8]. Murine β-defensin 2 has chemo-attractant activity for attracting immature dendritic cells, and can act as an endogenous ligand for TLR4 to promote the maturation of immature dendritic cells [9]. Given that dendritic cells are potent antigen presenting cells their activation should promote T cell immunity. Indeed, previous studies have shown that vaccination with the plasmids encoding for low immunogenic lymphoma antigen with β-defensin 2 or with β-defensin 2-expressing inactivated leukemia cells induces strong T cell immunity against lymphoma and leukemia, respectively [10], [11]. Furthermore, gene therapy with plasmid encoding for the mFLK-1 antigen and β-defensin 2 effectively inhibits the angiogenesis and growth of tumor *in vivo* [12]. However, whether β-defensin 2 could modulate melanoma antigen-induced NK and T cell responses has not been explored.

In this study, we employed a mouse model of melanoma, and tested the hypothesis that β-defensin 2 could act as an adjuvant to augment potent anti-melanoma immunity and inhibit the development and progression of melanoma *in vivo*. We first cloned the mouse β-defensin 2 gene by RT-PCR from mouse kidney tissue and generated stably β-defensin 2-expressing B16-mBD2 cells. Subsequently, we tested whether vaccination with sublethal-irradiated B16-mBD2 cells could augment potent NK activity and T cell immunity against melanoma in C57BL/6 mice. We found that vaccination with irradiated B16-mBD2, but not with control B16 or B16-p cells, inhibited the development and progression of melanoma and prolonged the survival of tumor-bearing mice, which was accompanied by inducing potent NK activity, melanoma-specific CTL responses, and IFN-γ and IL-12 production as well as CD8^+^ T, CD4^+^ T, macrophage and NK cell infiltration in the tumor tissues. Therefore, our data suggest that β-defensin 2 can act as an adjuvant to augment anti-melanoma immunity *in vivo*. We discussed the implications of our findings.

## Materials and Methods

### Cell lines

The C57BL/6-originated murine melanoma B16-F10 (designated as B16 here) cell line was a generous gift from Dr. Li-jing Wang (Institute of Basic Medical Sciences, Guangdong Pharmaceutical University, Guangzhou, China). Although B16-F10 cells were described as MHC-I negative [13], a low percentage of B16-F10 cells maintained in our lab were positive for anti-Kb and anti-Db staining (see below). Murine lymphoma Yac-1 cell line and hepatic carcinoma Hepa 1–6 cell line were purchased from the Cell Bank of Zhong-Shan Medical University, Guangzhou, China. All cell lines were cultured in RPMI 1640 supplemented with 10% fetal bovine serum (FBS), 100 Units/mL of penicillin, and 100 µg/mL of streptomycin at 37°C in a humidified 5% CO_2_ atmosphere.

### Animals

Female C57BL/6 mice at 6–8 weeks of age were purchased from the Center for Experimental Animals, Guangdong Province (Guangzhou, China). The mice were housed at a specific pathogen free facility of the Guangdong Provincial Key Laboratory of Pharmaceutical Bioactive Substances. All studies involving mice were approved by the Southern Medical University Animal Care and Use Committee (permission No. 2009-089).

### Plasmid construction

The murine β-defensin 2-expressing plasmid, pcDNA3.1**(+)**-Igκ-mBD2, was constructed. Briefly, total RNA was extracted from C57BL/6 mouse kidney using Trizol (Invitrogen) and reversely transcribed into cDNA using the SuperScript® VILO™ cDNA Synthesis kit (Invitrogen), according to the manufacturer's instruction. The gene for murine mature β-defensin 2 was amplified by PCR using the cDNA as the template and the specific primers. The sequences of primers were forward: 5′-GAACTTGACCACTGCCACACC-3′ and reverse: 5′-GCTCTAGATTATCATTTCATGTACTTGCAAC-3′. Subsequently, the DNA fragment was fused with a sequence for murine Igκ signal peptide by overlap-PCR using the following primers corresponding to mouse Igκ signal peptide sequence (63 bp) and 21 bp of annealing to the mature β-defensin 2 coding region. Recognition sequences of *BamH I* and *Xba I* were added to the sense and antisense primers, respectively. The sequences of all primers were sense 1: 5′-CTGCTCTGGGTTCCAGGTTCCACTGGTGACGAACTTGACC ACTGCCACACC-3′; sense 2:5′-CGGGATCCATGGAGTCAGACACACTCCTGCTATGG GTACTGCTGCTCTGGGTTCCAGGTTCC-3′; and antisense primer: 5′-GCTCTAGATTA TCATTTCATGTACTTGCAAC-3′. The PCR products were digested with *BamH I* and *Xba I,* and then cloned into the same sites of pcDNA3.1(+) vector, generating the plasmid of pcDNA3.1(+)-Igκ-mBD2. Its authenticity was demonstrated by DNA sequencing.

### Transfection of B16 cells

B16 cells at 6×10^5^/well were cultured overnight in 10% FBS RPMI 1640 (complete medium) in six-well plates and transfected with 4.0 µg/well of pcDNA3.1(+)-Igκ-mBD2 or control pcDNA3.1(+), respectively, using Lipofectamine2000 reagent (Invitrogen), according to the manufacturer's instruction. After being cultured for 24 h, the cells were treated with 0.5 mg/mL G418 (Merck, Germany) in complete medium for 4 weeks. Individual G418-resistant clones (B16-mBD2) were isolated and screened for the expression of β-defensin 2. A similar procedure was used for the generation of control B16-p clone that had been transfected with control plasmid pcDNA3.1(+).

### Expression of β-defensin 2

Individual transfectants at 10^6^ cells/mL were cultured for 72 h, and the cells and their supernatants were harvested. The levels of β-defensin 2 mRNA transcripts and β-defensin 2 proteins secreted in the supernatants were determined by RT-PCR and Western blot assays, respectively. Briefly, total RNA was extracted from the collected cells with Trizol, and reversely transcribed into cDNA. The levels of β-defensin2 mRNA transcripts were determined by PCR using the sense and anti-sense primers described above. The supernatants were separated by SDS-PAGE using 16.5% Tris-Tricine running gel and transferred electrically onto PVDF membrane (Millipore, Bedford, USA). After being blocked with 5% non-fat milk in TBST [150 mmol/L NaCl, 10 mmol/L Tris, and 0.05% Tween 20 (pH 8.0)] for 2 h at room temperature, the membranes were incubated overnight with 1∶400 diluted goat polyclonal IgG antibodies against murine β-defensin 2 protein (Santa Cruz, USA) in TBST at 4°C, and the bound antibodies were detected with horseradish peroxidase (HRP)–conjugated rabbit anti-goat IgG in TBST for 2 h at room temperature. The immunocomplex was visualized by 3,3′-diaminobenzidine (DAB).

### *In vivo* immunization studies

C57BL/6 mice were vaccinated subcutaneously (s.c) in right armpit with 1×10^6^ sublethal-irradiated B16-mBD2 cells, B16-p, or parental B16 cells for the induction of B16-specific immunity, respectively. One week after immunization, the mice were inoculated s.c with 5×10^4^ parental B16 cells in left armpit. The mice that had been injected with saline and inoculated with B16-p or B16 cells were used as controls.

To evaluate the therapeutic effect of vaccination with B16-mBD2 cells, C57BL/6 mice were inoculated s.c with 10^5^ parental B16 cells in left armpit, and the mice were injected in right armpit with saline, or treated s.c with 10^6^ sublethal-irradiated B16-mBD2 cells, B16-p cells, or parent B16 cells on day 0 and repeated three times on day 3, 7, and 11, respectively. The tumor sizes was measured with a caliper every other day up to 20 days post inoculation in a blinded fashion, and the tumor volumes were determined by the following formula: tumor volume (mm^3^) = π/6×length (mm)×width (mm)×width (mm) [14]. The survival of individual mice was recorded up to 150 days post inoculation.

To determine the role of CD4^+^ T and CD8^+^ T cells in the vaccination-related therapy, CD4^+^ and CD8^+^ T cells were depleted by treatment with the specific antibodies, respectively, as described previously [15], [16]. Briefly, groups of C57BL/6 mice were treated intraperitoneally with 200 µg/mouse of anti-CD4 monoclonal antibody (GK1.5), anti-CD8α monoclonal antibody (53–6.7) or rat IgG (eBioscience, San Diego, CA) daily on day −3, −2, −1, +6, +13, and +20, respectively. Three mice from each group were sacrificed on day 0 and the efficacy of T cell depletion was verified by flow cytometry analysis. Individual mice from those groups were inoculated s.c with 10^5^ B16 cells in left armpit and vaccinated with irradiated B16-mBD2 in right armpit on day 0. The mice were vaccinated with irradiated B16-mBD2 for three more times on day 3, 7, and 11 as described above. Additional groups of mice without antibody injection that had been inoculated with B16 cells were vaccinated with B16-mBD2 or injected with saline at each time point and used as positive or negative controls for therapeutic studies, respectively. The tumor sizes and the kinetics of tumor growth *in vivo* were measured and analyzed as described above. The survival of individual mice was monitored up to 120 days post inoculation.

### CTL and NK cell assays

Splenic mononuclear cells were prepared from individual mice one week after immunization. The cells were cultured at 37°C for 2 h and non-adherent cells were used as NK effector cells. To prepare CTL effector cells, splenic mononuclear cells (5×10^6^) were co-cultured with 5×10^5^ of mitomycin C (50 µg/mL, 40 min) inactivated B16 cells in 5 mL RPMI 1640 containing 10% FBS and 20 units/mL of recombinant IL-2 (Peprotech, Britain). Five days later, the cells were harvested, purified by Ficoll gradient centrifugation and used as CTL effector cells. The NK and CTL-mediated cytotoxicities were determined by lactate dehydrogenase (LDH) release assay. Briefly, Yac-1 cells at 2×10^4^/well were cultured in quadruplicate with the prepared NK at the ratios of 12.5, 25, and 50 effector to target cells, respectively in 5% FBS phenol-free RPMI 1640 in 96-well plates at 37°C for 4 h. The target cells or effector NK cells cultured in medium alone (negative) or treated with 1% triton X-100 (positive) were used as controls, respectively. After centrifugation, their supernatants were harvested and the contents of LDH were determined using the CytoTox96 Non-Radioactive Cytotoxixity Assay kit, according to the manufacturer's instructions (Promega, Madison, USA). The similar procedures were performed for CTL assays using B16 or irrelevant syngeneic control hepa 1–6 cells as targets, respectively. The cytotoxicity was calculated by the formula:
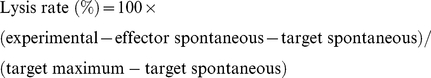



### Cytokine ELISA

Seven days post-immunization, splenic mononuclear cells were isolated from individual mice, and the splenocytes (5×10^6^/well) were challenged with mitomycin C (50 µg/mL, 40 min) inactivated B16 cells (5×10^5^/well) in 10% FBS RPMI1640 in the presence of 20 units/mL of IL-2 in 24-well plates for 48 h. Subsequently, their supernatants were harvested and the contents of IFN-γ, IL-12, and IL-4 were determined by ELISA using mouse IFN-γ, IL-12, and IL-4 ELISA kits, according to the manufacturer's instructions (R&D Systems, Minneapolis, USA).

### Histological examination

On day 30 post-inoculation, subcutaneous tumor nodules were dissected out from some mice of each group and fixed in 10% Formalin solution, dehydrated, and embedded in paraffin. The tumor tissue sections (5 µm) were stained with hematoxylin and eosin (H&E), and examined under a light microscope.

### Flow cytometry

On day 30 post-inoculation, subcutaneous tumor nodules were dissected out from some mice of the B16-mBD2-vaccinated group and the contents of CD4^+^, CD8^+^ T cells, NK cells, and macrophages in the tumors were characterized by flow cytometry. Briefly, the tumor tissues were minced small pieces and digested with 0.05 g/L of collagenase IV (Roche, Indianapolis, IN) and 0.05% of DNase I (Roche) at 37°C for 45 min, followed by passing through a stainless steel mesh (200 µM). After washing with D-Hanks, mononuclear cells were separated by Ficoll density gradient centrifugation. Subsequently, the cells were stained with anti-CD8-FITC, anti-CD4-FITC, anti-CD3-PE, anti-NK1.1-PE,anti-CD3-FITC, anti-F4/80-FITC and isotype control, respectively. The frequency of CD4^+^, CD8^+^ T cells, NK cells and macrophages was characterized by flow cytometry analysis on a FACScan using the CellQuest software (Becton Dickinson, San Jose, USA).

Additional flow cytometry analysis was used for characterizing the levels of H-2Kb/H-2Db on B16 cells using anti-H-2Kb-PE, and anti-H-2Db-PE, respectively. Briefly, B16 cells were stimulated with, or without, 350 pg/mL of IFN-γ for 4 or 24 h, respectively, and the cells were stained with anti-H-2Kb-PE, anti-H-2Db-PE and isotype controls, respectively, followed by flow cytometry analysis.

### Statistical analysis

Data are expressed as means ± SD or representative photoimages. The differences in individual measures among different groups were analyzed by ANOVA and the survival curves were plotted using Kaplan-Meier method and analyzed using the standard Mantel-Cox log-rank test. Differences were considered significant when a value of *P*<0.05.

## Results

### Cloning of the murine β-defensin 2 gene and characterization of murine β-defensin 2 expression

**Figure 1:**
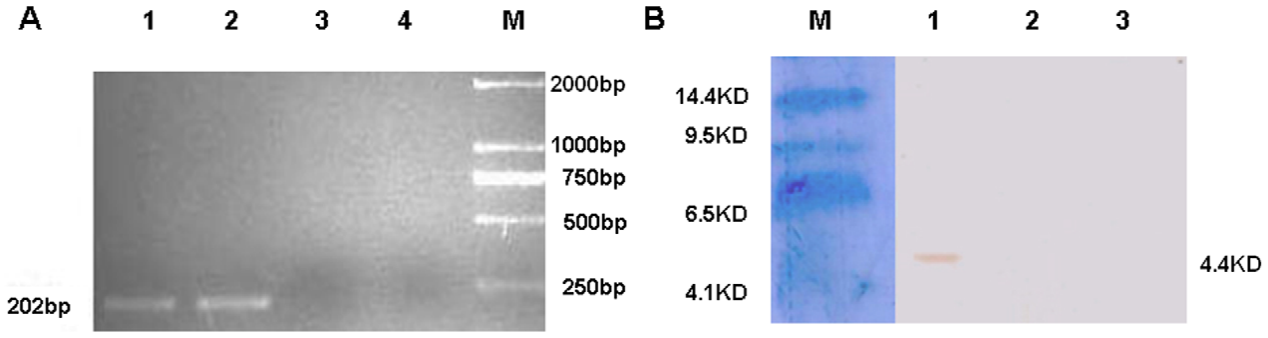
The expression of β-defensin 2 in B16-mBD2 cells. B16 cells were transfected with the plasmid, pcDNA3.1(+)-Igκ-β-defensin 2, or control pcDNA3.1(+), respectively, and treated with G418 for the generation of β-defensin 2 stably-expressing cell clones. The expression of β-defensin 2 was determined by RT-PCR and Western blot assays. (A) RT-PCR detection of β-defensin 2 mRNA. 1: The positive control plasmid containing Igκ-β-defensin 2 insert; 2: The pcDNA3.1(+)-Igκ-β-defensin 2 transfected B16 cells; 3: The pcDNA3.1(+) transfected B16 cells; 4: The parental B16 cells; M: DNA Marker. (B) Western-blot analysis of secreted β-defensin 2 peptides. M: protein Marker; 1: The supernatants of B16-mBD2; 2: The supernatants of B16-p; 3: The supernatants of parental B16. Data shown are representative images from three separate experiments.

To determine the role of β-defensin 2 in antitumor immunity, the murine β-defensin 2 gene was amplified from the cDNA of mouse kidney tissue by PCR. Subsequently, the DNA fragment was fused with a sequence for Igκ signal peptide and cloned into plasmid pcDNA3.1(+), generating the plasmid pcDNA3.1(+)-Igκ-mBD2. After being sequenced, pcDNA3.1(+)-Igκ-mBD2 and control pcDNA3.1(+) were transfected into B16 cells, respectively. Following G418 selection, the pcDNA3.1(+)-Igκ-mBD2-transfected B16-mBD2 and control plasmid-transfected B16-p were characterized for the expression of β-defensin 2 by RT-PCR, respectively (Fig. 1A). Obviously, while there was no detectable β-defensin 2 mRNA transcript in the parent B16 and the pcDNA3.1(+)-transfected B16-p cells, a clear visible band was detected in the B16-mBD2 cells. Hence, transfection of pcDNA3.1(+)-Igκ-mBD2 induced the transcription of β-defensin 2 mRNA in B16-mBD2 cells.

Further analysis of the supernatants of the cultured B16-mBD2, B16-p, and parent B16 cells by Western blot assays revealed that the β-defensin 2 protein was detected only in the B16-mBD2 cells, but not in B16-p and parent B16 cells (Fig. 1B). Together, these data indicated that the B16-mBD2 cells secreted β-defensin 2 *in vitro*.

### Vaccination with irradiated B16-mBD2 cells inhibits the growth of melanoma *in vivo*

**Figure 2:**
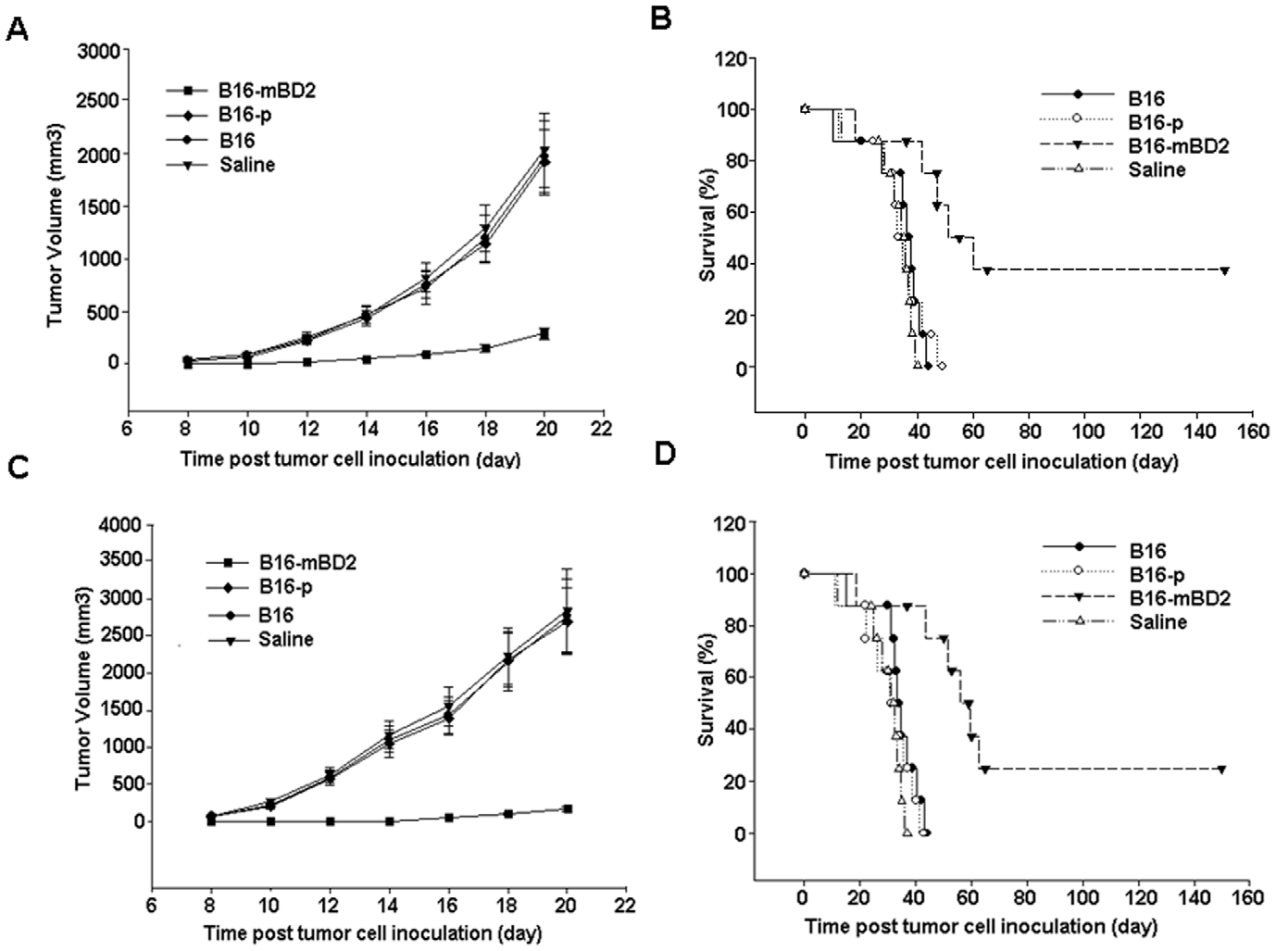
Vaccination with irradiated B16-mBD2 inhibits the growth of implanted tumors and prolonged the survival of mice. (A, B) The preventive effect of B16-mBD2 vaccination on the development of tumors. Groups of C57BL/6 mice were injected with saline or vaccinated with irradiated parental B16 cells, B16-p cells, or B16-mBD2 cells, respectively, and the mice were inoculated s.c with B16 tumor cells one week later. The growth of tumors in different groups of mice were monitored up to 20 days post inoculation (n = 8 per group), and their survival was monitored up to 150 day post inoculation (n = 8 per group). Data are a representative of three separate experiments and expressed as mean ± SD of the tumor volumes of each group of mice. (C, D) The therapeutic effect of vaccination with irradiated B16-mBD2 on the progression of implanted tumors. Groups of C57BL/6 mice were inoculated s.c with B16 cells, and then injected with saline or treated with irradiated parental B16 cells, B16-p cells, or B16-mBD2 cells for four times, respectively. The growth of tumors were monitored (n = 8 per group), and their survival were monitored up to 150 day post-treatment (n = 8 per group). Data are a representative of three independent experiments and expressed as mean ± SD of the tumor volumes of each group of mice.

β-defensin 2 is a immune regulator of innate and adaptive immunity, and may promote antigen-specific T cell immunity against malignant tumors [7]. To determine whether β-defensin 2 could enhance melanoma-specific T cell immunity, groups of C57BL/6 mice were injected with saline or vaccinated with sub-lethal irradiated B16-mBD2, B16-p, and patent B16 cells, respectively. Seven days after vaccination, individual mice were challenged s.c with 5×10^4^ of living parent B16 cells to establish solid tumors, and the growth of melanoma was monitored (Fig. 2A). The growth of solid melanoma in the mice vaccinated with irradiated B16 or B16-p cells was indistinguishable from that of the control mice received saline injection. In contrast, the growth of melanoma in the mice that received B16-mBD2 vaccination was significantly slow. On day 20 post inoculation, the mean tumor volume in the mice that had been vaccinated with irradiated B16-mBD2 was about 16% of that in the mice injected with saline or vaccinated with irradiated B16 cells. Furthermore, all mice in the saline, B16-p, and parental B16 groups died within 49 days post inoculation, while more than 60% of the mice vaccinated with B16-mBD2 survived. About 40% of the mice remained alive independent of tumor at the end of the 150-day observation period (P<0.01, by log-rank test, Fig. 2B). Apparently, vaccination with irradiated B16-mBD2 cells prevented the development of melanoma in mice.

Next, we examined whether vaccination with B16-mBD2 cells could therapeutically inhibit the development of melanoma *in vivo*. C57BL/6 mice were inoculated s.c with 10^5^ of living parent B16 cells on day 0 and randomly injected with saline or treated with 10^6^ of irradiated B16-mBD2, B16-p, and parent B16 cells on day 0, 3, 7, and 11, respectively. The development and progression of melanoma were monitored (Fig. 2C). Clearly, treatment with irradiated B16-mBD2 cells significantly inhibited the development and progression of melanoma in mice. All of the mice that received saline injection or irradiated B16-p, and parental B16 cells died within 44 days post-inoculation. In contrast, more than 80% of mice in the B16-mBD2 group remained alive on day 44 post inoculation, and nearly 30% of the mice remained alive free of tumor at the end of 150-day observation period (P<0.01, by log-rank test, Fig. 2D). Therefore, treatment with irradiated B16-mBD2, but not B16-p and parental B16 cells, inhibited the progression of melanoma and prolonged the survival of mice.

### Vaccination with irradiated B16-mBD2 induces NK and CTL responses and augments IL-12 and IFN-γ production

**Figure 3:**
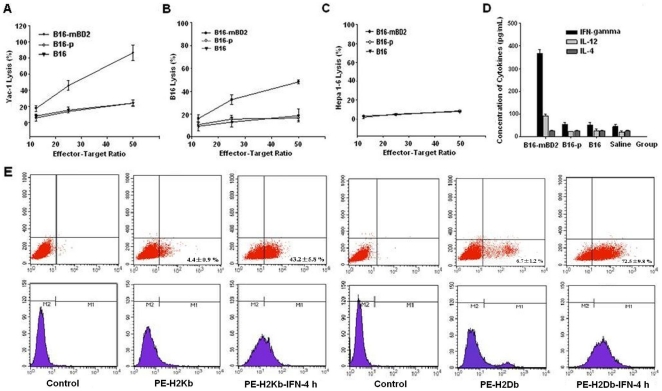
Vaccination induces NK, CTL and cytokine responses. (A) NK activity. Groups of C57BL/6 mice were vaccinated with irradiated B16-mBD2, B16-p, or B16, respectively, and one week later, their splenic mononuclear cells were isolated and used as NK effector cells. The NK effector cells were mixed with Yac-1 cells at the indicated ratios for four hours, and the cytotoxicity of NK effectors against Yac-1 was determined by LDH assays. (B, C) CTL response. Additional splenic mononuclear cells were challenged with inactivated B16 cells *in vitro*, and the lymphocytes were purified and used as CTL effectors. The cytotoxicity of CTL effectors against B16 (B) or syngeneic irrelavant control Hepa1–6 (C) was determined by LDH assays. Data are expressed as mean % ± SD of each group of mice (n = 3) from three independent experiments. (D) Cytokine responses. The supernatants of cultured mixture of splenic mononuclear cells and inactivated B16 cells were harvested, and the concentrations of indicated cytokines were determined by ELISA assays. Data are expressed as mean ± SD of each group of mice (n = 3) from three separate experiments. (E) Spontaneous and inducible MHC-I expression in B16 cell. B16 cells were treated with, or without, 350 pg/mL of IFN-γ for 4 or 24 h, respectively and the levels of H-2K^b^, H-2D^b^ expression on B16 cells were characterized by flow cytometry analysis. Data shown are representative histographes of each group of cells from five separate experiments. Much more frequent of Kb^+^ and Db^+^ B16 cells were observed following treatment with IFN-γ for 24 h (data not shown).

To examine the mechanisms underlying the anti-melanoma activity of irradiated B16-mBD2 vaccine, naive C57BL/6 mice were vaccinated s.c. with 1×10^6^ irradiated B16-mBD2, B16-p, or B16 cells, respectively. Seven days after vaccination, their splenic mononuclear cells were isolated and cultured for 2 h, the unadhered cells were used as NK effector cells. Additional splenic mononuclear cells were stimulated with inactivated B16 cells in the presence of IL-2 for 5 days, and lymphocytes were purified and used as CTL effectors. The NK-mediated cytotoxicity against Yac-1 cells and the CTL-mediated cytotoxicity against B16 cells or control Hepa1–6 cells were determined by LDH assays. While low levels of splenic NK cell activity were observed in the mice vaccinated with B16-p or parental B16, the levels of splenic NK activity against Yac-1 cells in the mice vaccinated with irradiated B16-mBD2 increased by 2–4 folds (Fig. 3A). Similar patterns of B16-specific splenic CTL responses were observed among these groups of mice (Fig. 3B). However, there were similar weak CTL responses to irrelevant syngeneic Hepa1–6 cells among these groups of mice (Fig. 3C). Apparently, vaccination with irradiated B16-mBD2, but not B16-p and parental B16, induced potent NK and CTL responses in mice.

Activated NK and T cells as well as antigen presenting cells can secrete pro-inflammatory cytokines, which promote and regulate cellular immunity against tumors. Next, we analyzed B16-specific IFN-γ, IL-12, and IL-4 production *in vitro*. Splenic mononuclear cells were isolated from individual groups of mice that had been vaccinated with irradiated B16-mBD2, B16-p, parental B16, or were injected with saline seven days after immunization and stimulated with inactivated B16 cells *in vitro* for 48 h. The concentrations of IFN-γ, IL-12, and IL-4 in the harvested supernatants were determined by ELISA (Fig. 3D). While similar levels of IL-4 were detected among different groups of mice, the levels of IFN-γ and IL-12 in the supernatants of cultured splenic mononuclear cells from the mice that received irradiated B16-mBD2 cells were elevated by 4–7 folds, as compared with other groups of mice. These data clearly indicated that vaccination of mice with irradiated B16-mBD2 augmented the production of IFN-γ and IL-12, but not IL-4.

Given that CTL responses depend on MHC-I expression and vaccination with B16-mBD2 induced high levels (about 350 pg/mL) of IFN-γ production *ex vivo*, we examined whether IFN-γ secreted by activated T cells could enhance MHC-I expression on B16. B16 cells were treated with, or without, 350 pg/mL of IFN-γ for 4 or 24 h and the levels of H-2Kb and H-2Db expression on B16 cells were characterized by flow cytometry analysis. As shown in Fig. 3E, we detected only 4.4±0.9% and 6.7±1.2% of H-2Kb^+^ and H-2Db^+^ B16 cells in the absence of IFN-γ, and 43.2±5.8% and 72.5±9.8% of H-2Kb^+^ and H-2Db^+^ B16 cells following stimulation with IFN-γ for 4 h, respectively (Fig. 3E, n = 5). More frequent of H-2Kb^+^ and H-2Db^+^ B16 cells were detected at 24 h post stimulation (data not shown). These results demonstrated that low frequency of B16 cells spontaneously expressed MHC-I and IFN-γ enhanced MHC-I expression on B16 cells.

### Both CD4^+^ and CD8^+^ T are involved in the B16-mBD2 vaccine-induced antitumor immunity

**Figure 4:**
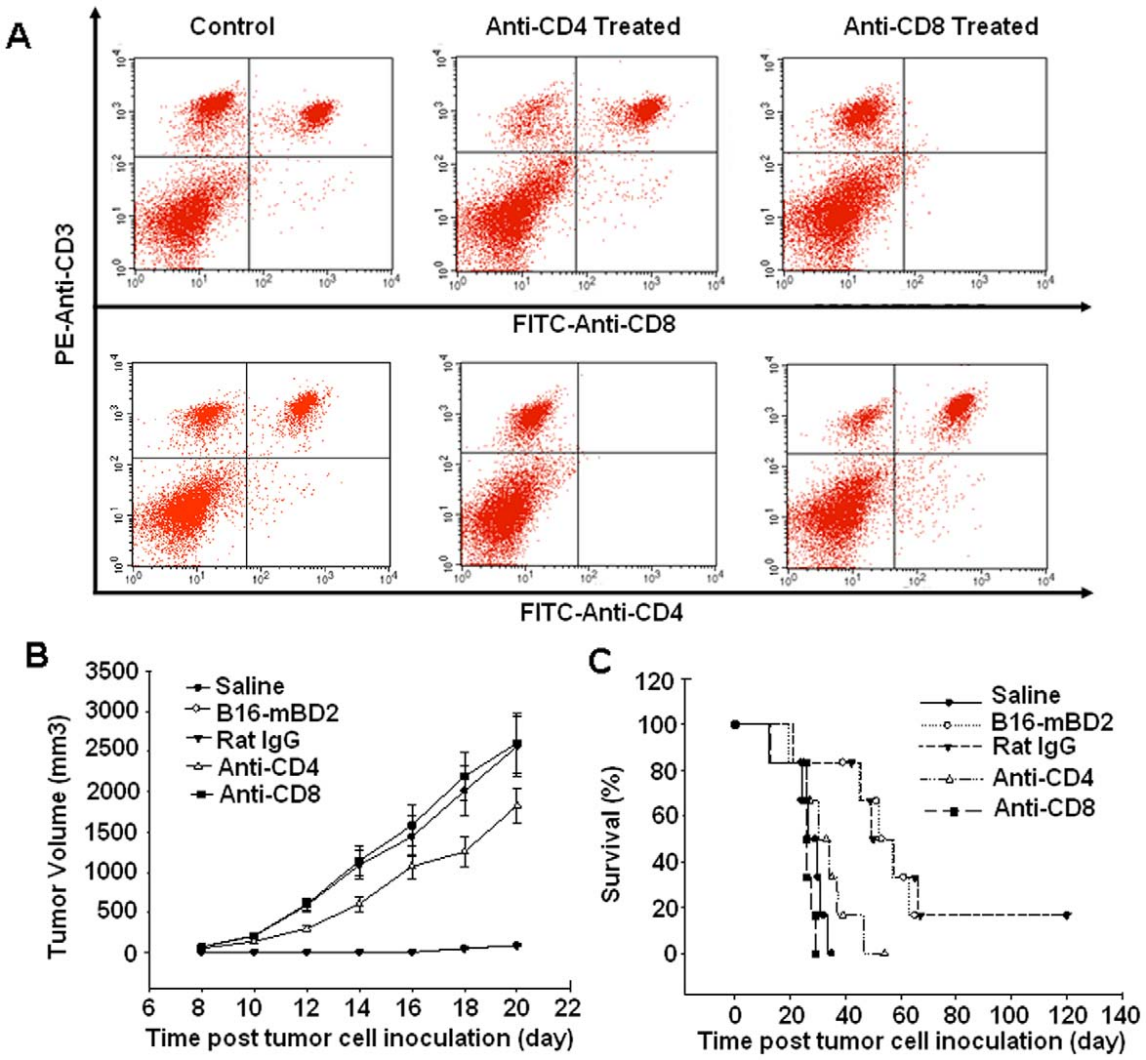
Pre-depletion of CD4^+^ or CD8^+^ T affects the therapeutic effect of B16-mBD2 vaccine in mice. (A) Flow cytometry analysis. Groups of C57BL/6 mice were injected with rat IgG, anti-CD4 or anti-CD8 antibody, respectively and three day after the initial antibody treatment, the frequency of splenic CD4^+^ and CD8^+^ T cells in some mice was characterized by flow cytometry analysis using FITC-anti-CD4, FITC-anti-CD8 and PE-anti-CD3. Data shown are representative images of each group (n = 3) of mice. (B, C) The effect of CD4^+^ or CD8^+^ depletion on the antitumor activity of irradiated B16-mBD2 vaccine. The remaining mice were continually injected with the corresponding antibody, inoculated with parental B16 cells in left armpit on day 0 and vaccinated with irradiated B16-mBD2 vaccine in right armpit on day 0, 3, 7, and 11. The growth of tumors was monitored (n = 3 per group), and their survival was monitored up to 120 days post inoculation (n = 6 per group). Data are expressed as mean % of survival rates of different groups of mice (n = 6 per group).

To assess the role of CD4^+^ or CD8^+^ T cells in the B16-mBD2 vaccination-induced antitumor immunity, groups of C57BL/6 mice were injected with control rat IgG, anti-CD4, or anti-CD8 monoclonal antibody to deplete CD4^+^ or CD8^+^ T cells and the frequencies of splenic CD4^+^ and CD8^+^ T cells were characterized by flow cytometry analysis three days after initial antibody treatment (Fig. 4A). Clearly, the frequency of CD4^+^ T cells in the anti-CD4-treated mice and CD8^+^ T cells in the anti-CD8-treated mice was reduced by more than 90%, demonstrating the successful depletion in these mice. Following inoculation of parent B16, vaccination with irradiated B16-mBD2 cells, and continual treatment with antibody, the growth of melanoma (Fig. 4B) and the survival of different groups of mice (Fig. 4C) were monitored. Tumor growth in the mice treated with anti-CD8 was indistinguishable from that of the mice injected with saline, but tumor growth in the mice treated with anti-CD4 was slightly slower than that of the mice treated with anti-CD8 and injected with saline. However, the tumor growth in the mice that had been vaccinated with B16-mBD2 and injected with rat IgG was significantly slower than that of the other three groups. Furthermore, we found that depletion of CD8^+^ or CD4^+^ T cells almost completely abolished the therapeutic effects of the B16-mBD2 vaccine (Fig. 4C). Evidentially, all of the mice that had been treated with anti-CD8, inoculated with B16 cells and vaccinated with B16-mBD2 died within 35 days post-inoculation, similar to that of saline-injected mice. In contrast, more than 80% of the mice in the B16-mBD2 therapeutic group and rat IgG control group remained alive on day 35 post inoculation, which was significantly longer than the saline treated control mice (P<0.01, by log-rank test). The mean survival period of mice that had been injected with anti-CD4 was significantly longer than that of the mice that had been injected with anti-CD8 (P<0.05, by log-rank test), but was much shorter than that of the rat IgG-treated and B16-mBD2 treated mice (P<0.05, by log-rank test, Fig. 4C). These data clearly indicated that both CD8^+^ and CD4^+^ T cells were the major players in the B16-mBD2-induced antitumor immunity, particularly for CD8^+^ T cells in this experimental model.

### Vaccination with irradiated B16-mBD2 promotes mononuclear cell infiltration in tumor tissues

**Figure 5:**
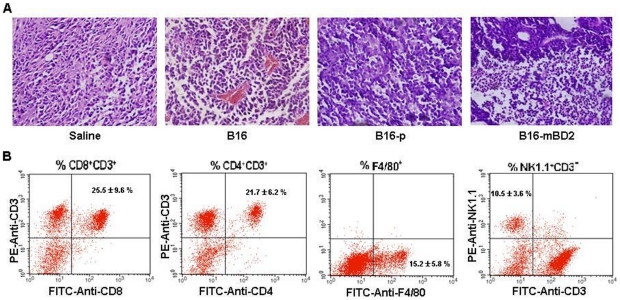
Vaccination with B16-mBD2 promotes macrophage, NK and T cell infiltration into the melanoma tumors. (A) Histological examination. On day 30 post inoculation, some tumor-bearing mice were sacrificed from each group and their tumors were dissected out, followed by H&E staining. Data shown are representative images (×400) from the indicated group of mice (n = 5). (B) Flow cytometry analysis of infiltrates. On day 30 post inoculation, some tumor-bearing mice were sacrificed from the B16-mBD2-vaccinated mice and their tumor tissues were dissected out, followed by digesting with collagenase and DNase I. Subsequently, mononuclear cells were isolated and stained with anti-F4/80-FITC, anti-CD4-FITC, anti-CD8-FITC and anti-CD3-PE, or anti-NK1.1-PE and anti-CD3-FITC, respectively, followed by flow cytometry analysis. Data shown are representative dot-plots or the mean % ± SD of CD8^+^CD3^+^, CD4^+^CD3^+^, F4/80^+^, NK1.1^+^CD3^−^ cells of five mice from three separate experiments.

Finally, we examined whether B16-mBD2 vaccination could modulate melanoma-related inflammation in the tumors. On 30 day post-inoculation, some mice from each group were sacrificed, and their tumor nodules were dissected out for histological examination. While there were a few mononuclear cells infiltrated in the tumors from the mice that received saline injection or vaccinated with B16 or B16-p cells, there were massive mononuclear infiltrates in the tumors from the mice vaccinated with B16-mBD2 (Fig. 5A). Next, we isolated mononuclear cells from freshly dissected tumors from the mice that had been vaccinated with irradiated B16-mBD2 and characterized the frequency of CD4^+^, CD8^+^ T cells, NK cells and macrophages by flow cytometry analysis. We found that 25.5±9.6% of CD8^+^ T, 21.7±6.2% of CD4^+^ T, 10.5±3.6% of NK cells and 15.2±5.8% of macrophages in the tumors from the B16-mBD2-vaccinated mice (Fig. 5B). These data suggested that vaccination with B16-mBD2 promoted the infiltration of CD8^+^ and CD4^+^ T cells, NK cells and macrophages in the tumors, an indicative of inflammation in the tumor tissues.

## Discussion

Effective antitumor immunity should recognize tumor antigens and destroy tumor cells *in vivo* [1]. However, many types of tumors in humans have low immunogenicity and require an adjuvant to induce an effective immune response. β-defensin 2 is an antimicrobial peptide and can regulate immune response [8]. Previous studies have shown that vaccination with plasmids for the fused protein of β-defensin 2 and tumor antigen or with β-defensin 2-expressing irradiated tumor cells stimulates strong antitumor immune responses against lymphoma, leukemia, and mammary adenocarcinoma *in vivo* [10], [11], [17], [18]. In this study, we determined whether β-defensin 2 could act as an adjuvant to induce melanoma-specific T cell immunity in mice. First, we cloned the gene for β-defensin 2 by RT-PCR and established the stable β-defensin 2-expressing B16-mBD2 cell clone by transfecting the β-defensin 2-expressing plasmid into B16 cells. Subsequently, we characterized the expression of β-defensin 2 and found that the β-defensin 2 mRNA transcripts were detected in B16-mBD2 cells, but not in control B16-p cells that had been transfected with control plasmid. Furthermore, we found that β-defensin 2 protein was detected in the supernatants of cultured B16-mBD2, determined by Western blot assays. These data suggested that β-defensin 2 was secreted by B16-mBD2 cells *in vitro*. The successful establishment of β-defensin 2-secreting B16-mBD2 cells provides a useful tool for further investigating the effect of vaccination with the β-defensin 2-expressing B16-mBD2 cells on the growth of implanted tumors *in vivo*.

Although sub-lethally irradiated tumor cells lack the ability to proliferate, they can survive for a short period and may continually secrete proteins. Indeed, we detected the expression of β-defensin 2 in cultured irradiated B16-mBD2 for at least four days post-irradiation, determined by RT-PCR (data not shown). Accordingly, we irradiated the B16-mBD2, control B16-p, and parental B16 cells, and tested whether vaccination with these cells could prevent and inhibit the growth of implanted tumors *in vivo*. This approach should vaccinate with majority of melanoma antigens and produce β-defensin 2 in mice for a short period. We found that pre-vaccination with irradiated B16-mBD2, but not with control B16-p or parental B16 cells, effectively inhibited the development of implanted melanoma in mice. More importantly, vaccination with B16-mBD2 cells on the same day with the implantation of melanoma cells also inhibited the development and progression of melanoma tumors in mice. In addition, vaccination with B16-mBD2 significantly prolonged the survival of mice that had been inoculated with parental B16 cells. Moreover, characterization of tumor tissues revealed many mononuclear cell infiltrates, including CD8^+^ and CD4^+^ T cells, NK cells and macrophages in the tumors from the mice that had been vaccinated with B16-mBD2, indicating that vaccination with B16-mBD2 induced a strong inflammation in the target tumors. These data suggest that the B16-mBD2 melanoma vaccine may be used for the therapeutic intervention of melanoma.

To understand the mechanisms underlying the protective effect of B16-mBD2 vaccination on the growth of implanted melanoma, we characterized the B16-mBD2-induced immune responses in mice. We found that high NK activity and strong CTL responses displayed in the mice vaccinated with B16-mBD2 cells, but not in the mice vaccinated with control cells. The vaccine-induced CTL responses appeared to be B16 melanoma-specific because similarly low CTL responses to unrelated Hepa1–6 cells were present in different groups of cells. Further analysis of the B16-stimualted cytokines revealed that higher levels of IFN-γ and significantly elevated levels of IL-12, but not IL-4, were secreted by splenic mononuclear cells from the mice vaccinated with B16-mBD2, but not other groups of mice. Possibly, vaccination with irradiated B16-mBD2 induced strong IFN-γ responses, which further up-regulated MHC-I expression on B16 cells, increasing their sensitivity to CTL responses. Indeed, we found that low frequency of B16 cells spontaneously expressed MHC-I and that the frequency of B16 cells expressing MHC-I dramatically increased following IFN-γ stimulation *in vitro*. Notably, depletion of CD4^+^ T cells before vaccination significantly decreased the therapeutic effect of B16-mBD2 vaccine while removing CD8^+^ T cells almost completely abrogated the B16-mBD2 vaccination-induced antitumor effect, suggesting that both types of T cells were important, but CD8+ T cells were the major mediators of the therapeutic effect of B16-mBD2 vaccine. These data are consistent with the notion that CD4^+^ T cells provide help in priming antitumor CD8^+^ CTL [19], [20]. Interestingly, the tumor growth and survival period of the CD4^+^ T cell-depleted mice was slightly slower and significantly longer than that of the CD8^+^ T cell-depleted mice. The slower tumor growth and the extended survival in the CD4^+^ T cell-depleted mice may stem from the CD4-independent activation of some CD8^+^ T cells [21], [22], which can inhibit the growth of implanted tumor cells. Alternatively, a few CD4^+^ T cells in the anti-CD4-treated mice may be sufficient in providing help for the activation of small population of CD8^+^ T cells. Moreover, characterization of tumor tissues revealed many mononuclear cells infiltrated in the tumors from the mice vaccinated with B16-mBD2, but not from other groups of tumors. Further characterization indicated that CD8^+^ and CD4^+^ T cells, NK cells, and macrophages were predominant infiltrates in the tumors from the B16-mBD2-vaccinated mice, indicating that vaccination with B16-mBD2 induced a strong inflammation in the target tumors. IFN-γ is predominately secreted by activated Th1, CD8^+^ T cells, and NK cells while IL-12 is produced by activated antigen presenting cells. Conceivably, vaccination with B16-mBD2 induces pro-inflammatory Th1, CD8^+^T cells, and activates NK cells, consistent with previous findings [11], [17]. As a result, the activated immunocompetent cells may migrate into the tumor tissues, inhibiting the growth of implanted melanoma in mice. Hence, our study provides additional evidence to support the notion that β-defensin 2 can act as an adjuvant to promote antitumor immunity *in vivo*. Notably, Th17 cell responses have been thought to be crucial for antitumor immunity. We are also interested in further investigating whether vaccination with B16-mBD2 could induce B16-specific Th17 responses in mice.

β-defensin 2 has chemotactic activity and can recruit immature dendritic cell migration through the receptor of CCR6 [7]. We speculated that β-defensin 2 was produced by the irradiated B16-mBD2 cells at least for couple days post-inoculation and it recruited dendritic cells and other immunocompetent cells through CCR6 into the vaccination site. Given that dendritic cells have potent antigen presenting activity, they can capture and present the apoptotic irradiated B16-mBD2 cell antigens to induce strongly pro-inflammatory T cell immunity, which inhibits the growth of implanted melanoma in mice. Apparently, β-defensin 2 acts as an adjuvant and provides a “danger signal” for stimulating pro-inflammatory T cell responses. Indeed, β-defensin 2 has been shown to promote the maturation of dendritic cells, which elicit potent antitumor immunity against breast cancers in mice [18]. Therefore, β-defensin 2 may be used as an adjuvant to stimulate pro-inflammatory T cell responses against tumors.

In summary, our data indicated that vaccination with the β-defensin 2-expressing irradiated B16-mBD2 cells inhibited the growth of implanted melanoma cells in mice. The therapeutic effects of B16-mBD2 vaccination were likely mediated by vaccine-induced strong pro-inflammatory NK and T cell immunity, particularly for CD8^+^ T cell responses, high levels of IFN-γ and IL-12 production and massive CD8^+^ and CD4^+^ T cell, NK and macrophage infiltration in the tumor tissues. Our findings suggest that β-defensin 2-expressing irradiated tumor cell-based vaccine is a promising strategy for the induction of effective antitumor immunity.
